# Assessing the causal relationship between genetically determined inflammatory biomarkers and low back pain risk: a bidirectional two-sample Mendelian randomization study

**DOI:** 10.3389/fimmu.2023.1174656

**Published:** 2023-07-13

**Authors:** Wenhan Li, Qunwen Lu, Junhui Qian, Yue Feng, Jian Luo, Caigui Luo, Wenshan He, Bing Dong, Huahui Liu, Zhongxing Liu, Chengguo Su

**Affiliations:** ^1^ Tui-Na Department, Hospital of Chengdu University of Traditional Chinese Medicine, Chengdu, Sichuan, China; ^2^ Department of Acupuncture, Moxibustion, Tui-Na and Rehabilitation, Guang'an City Hospital of Traditional Chinese Medicine, Guangan, Sichuan, China; ^3^ Tui-Na Teaching and Research Department, College of Acupuncture and Tuina, Chengdu University of Traditional Chinese Medicine, Chengdu, Sichuan, China; ^4^ Tui-Na Department, Meishan City Hospital of Traditional Chinese Medicine, Meishan, Sichuan, China; ^5^ Rehabilitation Department, School of Clinic Medicine & The First Affiliated Hospital of Chengdu Medical College, Chengdu, Sichuan, China; ^6^ Chinese Medicine Rehabilitation Department, Jiahekang Hospital, Luzhou, Sichuan, China; ^7^ Department of Acupuncture, Moxibustion, Tui-Na and Rehabilitation, The Affiliated Traditional Chinese Medicine Hospital of Southwest Medical University, Luzhou, Sichuan, China; ^8^ Center for Traditional Chinese Medicine Prevention and Health Care, Chengdu Integrated TCM & Western Medicine Hospital, Chengdu, Sichuan, China

**Keywords:** inflammatory biomarkers, low back pain, Mendelian randomization, causality, interleukin 6 (IL-6)

## Abstract

**Background:**

Observational studies have suggested an association between inflammatory markers and low back pain (LBP), but the causal relationship between these factors remains uncertain.

**Methods:**

We conducted a bidirectional two-sample Mendelian randomization analysis (MR) study to investigate whether there is a causal relationship between inflammatory markers and low back pain. We obtained genetic data for CRP, along with its upstream inflammatory markers IL-6, IL-8, and IL-10, as well as low back pain from publicly available genome-wide association studies (GWAS). We applied several MR methods, including inverse variance weighting, weighted median, MR-Egger, Wald Ratio, and MR-PRESSO, to test for causal relationships. Sensitivity analyses were also conducted to assess the robustness of the results.

**Results:**

Our analyses utilizing the Inverse Variance Weighted (IVW) method, the MR-Egger method, and the weighted median method indicated that IL-6 may be associated with an increased risk of LBP (Effect Size: -0.009, 95% Confidence Interval: -0.013–0.006, p = 9.16e-08); however, in the reverse direction, there was no significant causal effect of LBP on inflammatory markers.

**Conclusion:**

Our study used a Mendelian randomization approach and found that elevated IL-6 levels may reduce the risk of LBP.

## 1 Introduction

Low back pain (LBP) is a prevalent musculoskeletal disorder that has a profound impact on quality of life. In fact, it has been identified as the leading cause of disability and work-related absenteeism in 126 countries (1). This problem has become a significant public health issue, and it has a considerable economic burden on society (2, 3). In the United States alone, the direct and indirect costs associated with LBP surpass $100 billion annually, including medical expenses, lost wages, and decreased productivity (4). As the aging population continues to grow globally, the economic burden of LBP is expected to increase, underscoring the need to identify the risk factors for its development.

Inflammation is believed to be a key mechanism underlying LBP and spinal degeneration (3, 5). C-reactive protein (CRP) is a well-known biomarker of systemic inflammation, and previous observational studies have shown a positive correlation between its levels and the severity of LBP (6–8). Pro-inflammatory cytokines, such as interleukins, trigger the production of inflammatory markers like CRP in the liver (9). Several observational studies have found that patients with LBP have higher levels of serum IL-6 and IL-8 protein and mRNA compared to healthy controls (10–12). Additionally, IL-10 is considered an anti-inflammatory marker that inhibits the synthesis of pro-inflammatory cytokines. Observational studies have shown an inverse association between IL-10 and LBP pain severity (10, 12). However, conventional observational studies have limitations, and the association between inflammatory markers and LBP reported in previous studies may still be explained by reverse causality and residual confounding (13, 14). Therefore, the causal relationship between inflammatory markers and LBP remains uncertain.

Mendelian randomization (MR) is a widely used analytical method that employs genetic variation as an instrumental variable to produce more reliable causal estimates of the effect of risk factors on long-term exposure to disease outcomes (14, 15). The rationale for relying on MR as a more powerful method of causal inference than traditional observational studies stems from Mendel’s law and the fact that genotypes for germline genetic variation are determined at the time of conception and are often unrelated to the traditional confounders of observational studies. Previous studies have demonstrated the utility of genetic tools to elucidate the causal relationship between inflammatory markers and disease risk (16). Therefore, in this bidirectional two-sample Mendelian randomization analysis study, our aim was to investigate the causal relationship between CRP, as well as its upstream inflammatory markers IL-6, IL-8, and IL-10, and the risk of developing LBP.

## 2 Methods

### 2.1 Study design

**Figure 1:**
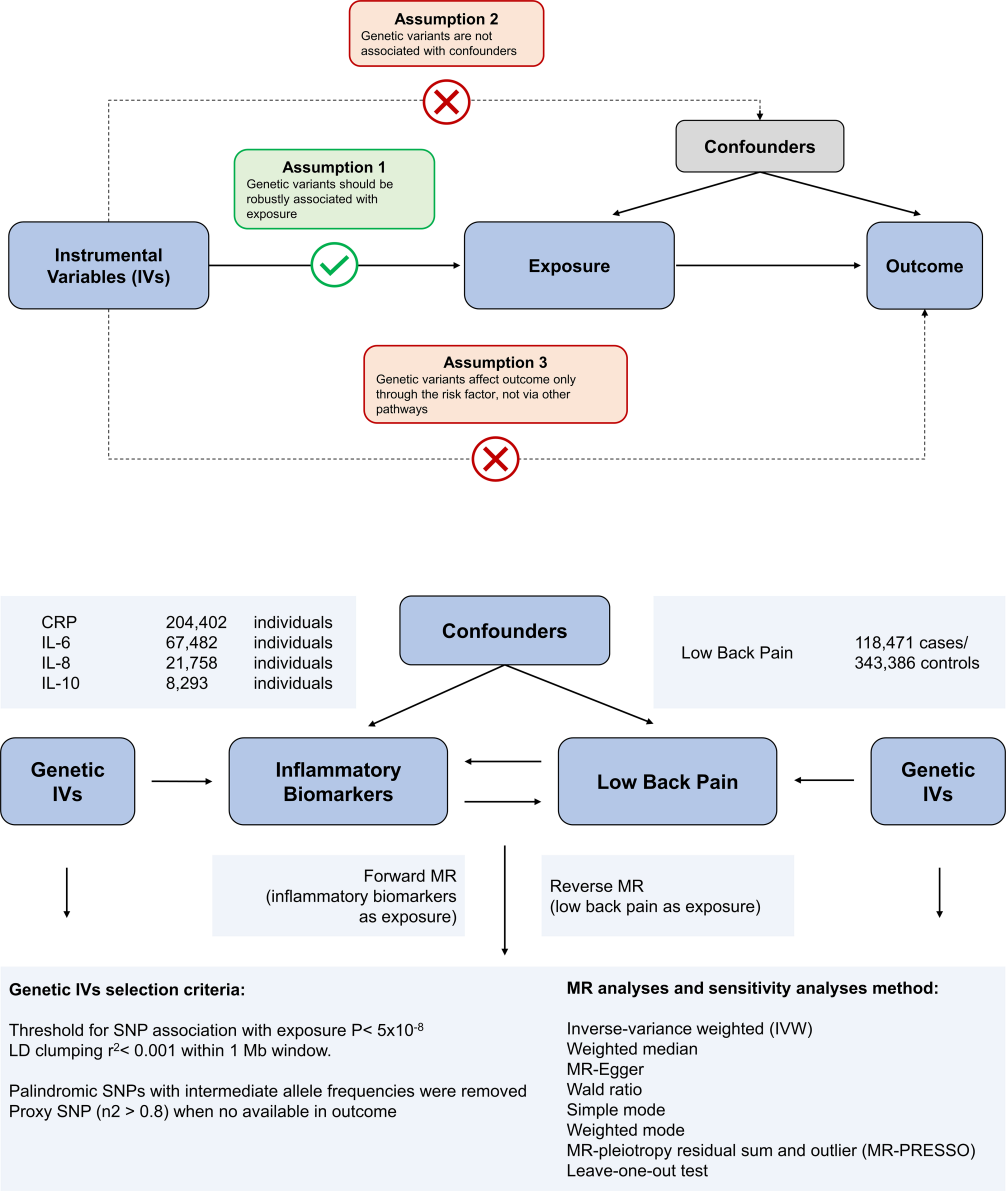
Overview of the assumptions of the Mendelian randomization (MR) design and the study design. The MR design reduces residual confounding and reverse causality, thereby strengthening causal inferences about exposure-outcome associations. This is because genetic variants used as instrumental variables to study altered exposure effects are randomly assigned at the time of conception and are therefore not susceptible to confounding by environmental factors or reverse causality.

Mendelian randomization is built upon three main assumptions. First, genetic variants are associated with the exposure. Second, there should be no association between genetic variants and confounders. Third, genetic variants influence the risk of the outcome only through the exposure, not through other pathways (17). In the present study, we employed bidirectional MR analysis to comprehensively infer the causal relationship between inflammatory markers and low back pain in both the forward and reverse directions. We have summarized our study design in **Figure 1**.

### 2.2 Data sources

The variants associated with CRP were obtained from a genome-wide association study (GWAS) of up to 204,402 individuals of European ancestry (18). The variants associated with IL-6 were obtained from a genome-wide association study (GWAS) of up to 67,482 individuals of European ancestry (19). Variants associated with IL-8 were derived from a GWAS of up to 21,758 individuals of European ancestry from the SCALLOP consortium (20). Genetic variants for IL-10 were collected from a GWAS of 8,293 Finnish individuals (21). The genetic association with back pain was obtained from the UK Biobank abstract data and can be found by searching for GWAS ID: ukb-b-9838 on the web at https://gwas.mrcieu.ac.uk/datasets/. The identified genetic variants were found to be associated with the self-reported experience of back pain within the past month. The phenotype was derived from responses to a touchscreen questionnaire, which asked participants: “In the last month, have you experienced any of the following symptoms that interfered with your usual activities? (You may select more than one answer).” The response options included: Headache, Facial pain, Neck or shoulder pain, Back pain, Stomach or abdominal pain, Hip pain, Knee pain, and Pain all over the body. Additional validation checks were performed to ensure the accuracy of the phenotype. Specifically, if a participant selected “Pain all over the body,” “Knee pain,” or “Neck or shoulder pain,” no additional response options were permitted. This GWAS includes 461,857 unrelated individuals of European ancestry, comprising 118,471 cases and 343,386 controls. To reduce selection bias and improve the robustness of the analysis, all datasets used in our study comprised individuals of European ancestry. All data used in this study were obtained from GWAS, with prior ethical approval and patient consent. The study protocol complied with the guidelines of the Declaration of Helsinki and was approved by the ethics committees of all participating sites. The details of these GWAS data sources are listed in **Supplementary Table S1**. There is no overlap between the exposure GWASs and the outcome GWASs.

### 2.3 Selection of genetic instruments

To identify SNPs for MR analysis, we selected SNPs associated with the exposure under genomewide significance threshold (p < 5x10^-8) as instrumental variables (IVs). These SNPs were further screened using a LD distance threshold of 10,000 kb and r^2^ < 0.001 to ensure independence between genetic variants (22). We calculated the F-statistic for each IV to assess the strength of its association with the exposure (23) and included IVs with an F-statistic > 10 (24). Subsequently, the palindromic SNPs were removed to ensure that their association effects were linked with the same alleles in both the exposure and outcome.

### 2.4 Statistical power calculation

We sought to assess the statistical power of our MR analyses through the use of an online web tool (https://sb452.shinyapps.io/power) (25). The assessment of statistical power for MR analyses was based on several parameters, including the total sample size, the significance level of 0.05/8, the proportion of variance (R2) in the exposure explained by instrumental variables, and the ratio of cases to controls.

### 25 Statistical analyses

To test for causal effects of exposure on outcome, we performed MR analyses using five methods, namely inverse variance weighting (IVW) (26), weighted median (27), MR-Egger (28), Wald Ratio(for IL-8, because only one SNP was available), and MR pleiotropic residuals and outliers (MR-PRESSO) (29). These five methods make different assumptions and use different strategies to deal with IVs with horizontal pleiotropy effects. The IVW method relies on the assumption that there is no pleiotropy and assumes that all SNPs are valid genetic tools (26). The weighted median method assumes that at least 50% of the IVs are valid (27). The MR-Egger method provides causal estimates even if all IVs are invalid (28). The MR-PRESSO method detects possible IV outliers by global testing and provides unbiased causal estimates by removing the identified outliers (29).

Heterogeneity in causal estimates among instrumental variables indicates a potential violation of the assumptions of MR analysis. The Cochran’s Q test was used to examine the heterogeneity in causal estimates, and we used both the causal estimates of fixed effects IVW method and MR-Egger regression to detect heterogeneity. The heterogeneities were quantified using Cochran’s Q statistics and a P-value smaller than 0.05 was considered significant heterogeneity. To assess the potential pleiotropic effects of instrumental variables, the MR-Egger regression was used. The directional horizontal pleiotropy in the causal estimates may be indicated by the intercept term in MR-Egger regression. Additionally, we performed a leave-one-out analysis where we excluded each SNP in turn and then ran MR analysis on the remaining SNPs in order to detect potentially outlying instrumental variables.

All results are expressed as effect size (ES) and corresponding 95% confidence intervals (CI). All statistical analyses were two-sided. A p-value was considered statistically significant when less than 0.006 (0.05/8 adjusted with the Bonferroni method) and was considered suggestively significant between 0.006 and 0.05. All analyses were performed using the TwoSampleMR and MR-PRESSO packages in R version 4.2.2.

## 3 Result

### 3.1 Causal effects of different inflammatory biomarkers on the risk of low back pain

To assess the impact of inflammatory markers on the risk of low back pain, we conducted an MR analysis. Initially, we identified 50, 1, 94, and 2 SNPs associated with CRP, IL-8, IL-6, and IL-10, respectively. The characteristics of these IVs are shown in **Supplementary Table S2–S5**. In addition, we calculated the statistical efficacy of each exposure in each cohort (CRP, 100%; IL-8, 83.3%; IL-6, 57.1%; IL-10, 4.3%) (**Supplementary Table S6**).

**Figure 2:**
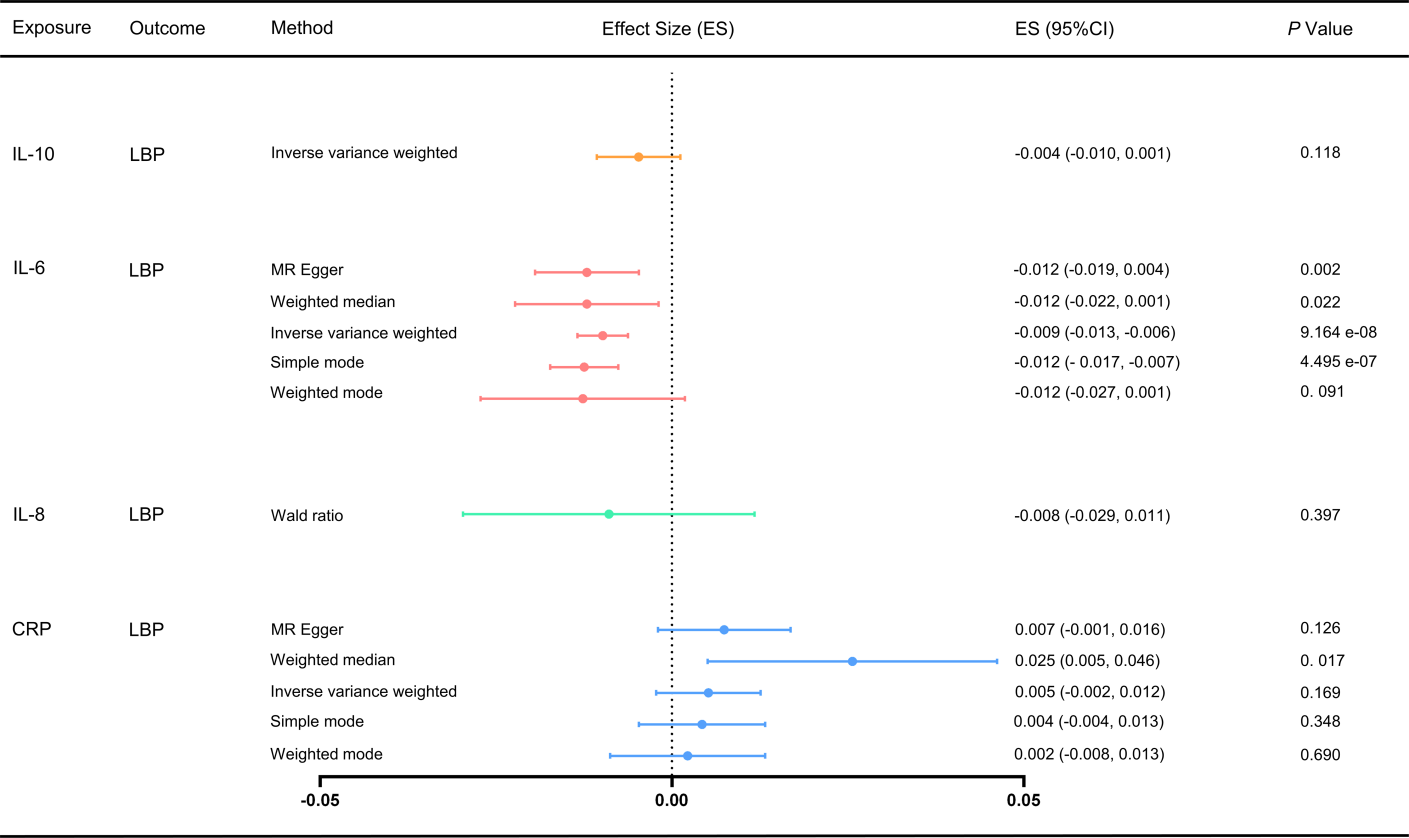
Effect of different inflammatory markers on LBP. IL-10 did not have enough SNP to complete the weighted median, MR-Egger and MR-PRESSO. MR, Mendelian randomization; MR-PRESSO, Mendelian randomization pleiotropy residual sum and outlier test; ES, effect size; CI, confidence interval.

We used the IVW and MR-Egger methods to evaluate the effect of different inflammatory markers on low back pain, and the results showed IL-6 may be associated with an increased risk of LBP (ES: -0.009, 95% CI: -0.013, -0.006, p = 9.164e-08), and no significant causal relationship between other inflammatory markers on low back pain (**Figure 2**; **Supplementary Table S6**). Furthermore, Cochran’s Q test showed no heterogeneity (CRP-LBP, Q =131.904, p = 1.141; IL-6 -LBP, Q =47.016, p = 0.999; IL-10-LBP, Q = 0.215, p = 0.642). We also conducted a horizontal pleiotropy test, which indicated no directional pleiotropy (CRP-LBP, intercept = 0.0002, p = 0.480; IL-6 -LBP, intercept = 0.0001, p = 0.694). The MR-PRESSO global test also showed no horizontal pleiotropy effects (CRP-LBP, p = 0.159; IL-6-LBP, p = 0.500). To ensure that the results were not influenced by a single SNP, we conducted a leave-one-out sensitivity test for IL-6, which demonstrated that the causal effect of IL-6 on LBP did not fluctuate significantly with the absence of any single SNP (**Supplementary Figure S1**).

### 3.2 Causal impact of low back pain on different inflammatory markers

**Figure 3:**
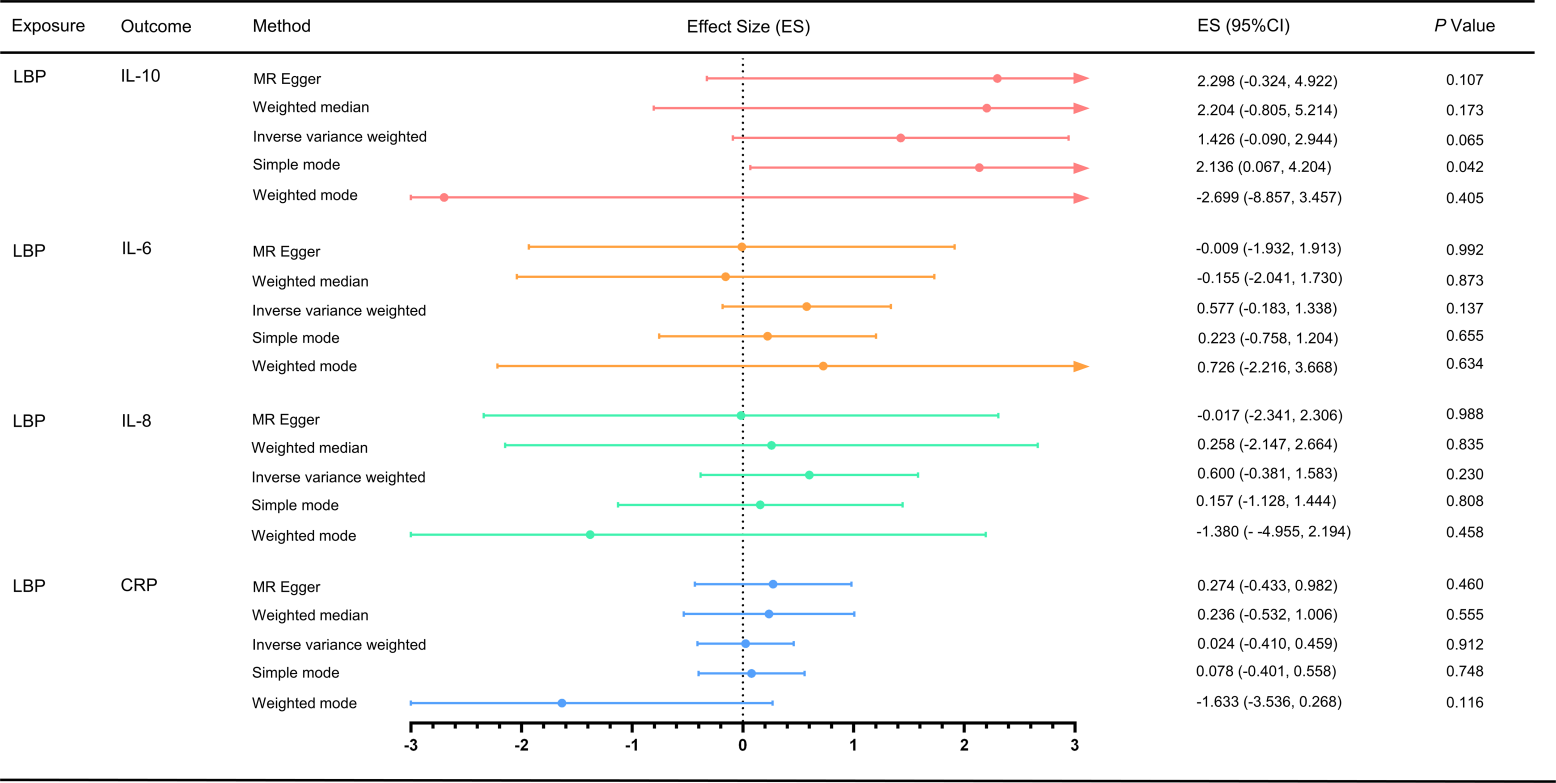
Effect of LBP on different inflammatory markers. MR, Mendelian randomization; MR-PRESSO, Mendelian randomization pleiotropy residual sum and outlier test; ES, effect size; CI, confidence interval.

For the MR analysis of low back pain with different inflammatory markers, we obtained SNPs associated with low back pain from the GWAS (**Supplementary Tables S7**-**S10**). We performed IVW analysis, MR-Egger method, weighted median method, and weighted mode method, but found no evidence of a causal effect of low back pain on inflammatory markers (p > 0.05) (**Figure 3**). Cochran’s Q test showed no heterogeneity (LBP-CRP, Q = 19.264, p = 0.115; LBP-IL6, Q = 8.536, p = 0.969; LBP-IL-10, Q = 9.637, p = 0.723; LBP-IL8, Q = 20.729, p = 0.293). All Egger regression tests were negative (LBP-CRP, intercept = 0.011, p = 0.103; LBP-IL6rα, intercept = -0.001, p = 0.919; LBP-IL10, intercept = 0.030, p = 0.198; LBP-IL8, intercept = 0.014, p = 0.273). The MR-PRESSO global test also showed no horizontal pleiotropy effects (LBP-CRP, p = 0.933; LBP-IL6rα, p = 0.360; LBP-IL10, p = 0.214; LBP-IL8, p = 0.422), indicating that our MR results were not influenced by horizontal pleiotropy. Since there are no positive results, leave-one-out sensitivity test are inapplicable here. In addition, we calculated the statistical efficacy for each outcome in each cohort. The results showed that the statistical efficacy of each exposure was 100%, thus confirming the robustness of our MR analysis. (**Supplementary Table S6**)

## 4 Discussion

In this study, we employed bidirectional MR analysis to investigate a potential causal relationship between different inflammatory factors and low back pain. Using the largest publicly available GWAS dataset, we found that lower IL-6 level was associated with higher risk of LBP; however, in the reverse direction, there was no significant causal effect of LBP on inflammatory markers.

Previous studies have demonstrated a strong association between them. For instance, CRP was the first inflammatory marker reported to be associated with low back pain. As early as 2005, an observational study found that CRP was linked to pain levels in patients with acute low back pain (6). IL-6 promotes the differentiation of monocytes into macrophages and activates lymphocyte maturation to help mediate the acute phase response to injury (30). Queiroz et al. found that IL-6 was associated with both the severity of LBP and disability due to LBP (31). A systematic review that included six studies found evidence of elevated CRP in patients with acute LBP, but not elevated IL-6 in patients with LBP (32). Another systematic review that included six studies found an association between elevated CRP and IL-6 and low back pain, supporting the idea that there is a positive correlation between inflammatory markers and low back pain (33). Wang et al. found higher serum IL-6 and IL-8 levels in patients with severe LBP than in mild and control subjects (12). Conversely, IL-10 is an anti-inflammatory marker, and Wang et al. found that patients with mild LBP had lower serum IL-10 levels than those with severe LBP (12). The levels of inflammatory markers were influenced by the degree of low back pain and time to relief, which may be the reason why this MR study did not find a causal effect of LBP on inflammatory biomarkers.

Previous studies have identified possible mechanisms by which inflammatory markers are involved in the development of LBP. Lumbar disc degeneration is an important cause of low back pain (34). Park et al. found that serum levels of IL-6 and IL-8 were higher in patients with lumbar disc herniation than in controls (35). Pedersen et al. found that chronic lumbar radicular pain may be associated with a sustained increase in serum pro-inflammatory substances IL-6 and IL-8 after disc herniation surgery (36). These inflammatory biomarkers are thought to promote matrix degradation, chemokine production, and cellular phenotypic changes. The resulting imbalance between catabolic and anabolic reactions leads to lumbar disc degeneration, disc herniation and nerve root pain (3).

Contrary to the findings of many previous observational studies, our results suggest that elevated levels of IL-6 may be associated with a decreased risk of LBP. This may be attributed to the potential role of an active inflammatory response in reducing the incidence of chronic low back pain. For instance, Marc et al. conducted a transcriptome-wide analysis of peripheral immune cells in 98 subjects with acute LBP and observed thousands of dynamic transcriptional changes over a 3-month period in subjects who experienced pain relief, but not in those with persistent pain (37). Their findings suggest that neutrophil-driven upregulation of the inflammatory response may prevent the transition to chronic pain in LBP patients. In a mouse pain assay, they also found that early treatment with steroids or non-steroidal anti-inflammatory drugs (NSAIDs) resulted in prolonged pain, whereas no such prolongation was observed with other analgesics. Furthermore, their analysis of pain trajectories in human subjects reporting acute back pain in the UK Biobank revealed an increased risk of pain persistence in subjects taking NSAIDs. Our study supports the findings of Marc et al., indicating that an active inflammatory response may prevent the transition to chronic low back pain in LBP patients. Additionally, the observed association between elevated IL-6 levels and a reduced risk of LBP may be explained by the fact that lumbar disc herniation is the most common cause of LBP. Weber et al. reported that serum IL-6 levels were significantly higher in patients with lumbar disc degeneration and spinal stenosis than in those with lumbar disc herniation (38).

To our knowledge, no MR studies have been reported on the causal effects of inflammatory markers on LBP or vice versa. Our study utilized multiple IVs from GWAS of inflammatory markers and LBP to increase statistical power for detecting causality, providing a more precise assessment of effect sizes.

However, our study has several limitations. Firstly, the limitations of Mendelian Randomization (MR) analysis preclude a thorough examination of the second and third assumptions and may introduce bias. For instance, several factors that contribute to low back pain may also contribute to inflammation, including mechanical trauma, obesity, and infection. Secondly, the population included in this MR analysis is of European descent and it remains to be determined whether the results can be replicated in Asian and African populations. Thirdly, we did not stratify the causal relationship between low back pain and inflammatory markers according to the duration of low back pain, despite evidence suggesting that the duration of low back pain can influence the levels of inflammatory markers in the blood. Fourth, it is important to note that the genetic association between IL-6 and IL-10 and LBP was based on a relatively small GWAS, resulting in limited statistical power (57.1% for IL6-LBP and 4.3% for IL10-LBP). Therefore, a subsequent GWAS with a larger sample size is necessary to confirm and update the findings of this study.

## 5 Conclusion

Our study used a Mendelian randomization approach and found that elevated IL-6 levels may reduce the risk of LBP.

## Data availability statement

The original contributions presented in the study are included in the article/**Supplementary Materials**. Further inquiries can be directed to the corresponding authors.

## Author contributions

Conceptualization, WL, ZL and CS; Methodology, HL, QL and CS; Formal analysis, WL, WH and JQ; Software, WL, JL and QL; Data Curation, WL and CS; Investigation, JL, YF and CL; Validation, CL, and YF; Resources, WL and YF; Visualization, WL and HL; Supervision, HL and ZL; Writing-original draft, WL, CS and QL; Writing-review and editing, CS and CL; Funding acquisition, CS; Project administration, WL and CS. All authors contributed to the article and approved the submitted version.
