# Corrigendum: Assessing the causal relationship between genetically determined inflammatory biomarkers and low back pain risk: a bidirectional two-sample Mendelian randomization study

**DOI:** 10.3389/fimmu.2023.1327338

**Published:** 2023-11-14

**Authors:** Wenhan Li, Qunwen Lu, Junhui Qian, Yue Feng, Jian Luo, Caigui Luo, Wenshan He, Bing Dong, Huahui Liu, Zhongxing Liu, Chengguo Su

**Affiliations:** ^1^ Tui-Na Department, Hospital of Chengdu University of Traditional Chinese Medicine, Chengdu, Sichuan, China; ^2^ Department of Acupuncture, Moxibustion, Tui-Na and Rehabilitation, Guang’an City Hospital of Traditional Chinese Medicine, Guangan, Sichuan, China; ^3^ Tui-Na Teaching and Research Department, College of Acupuncture and Tuina, Chengdu University of Traditional Chinese Medicine, Chengdu, Sichuan, China; ^4^ Tui-Na Department, Meishan City Hospital of Traditional Chinese Medicine, Meishan, Sichuan, China; ^5^ Rehabilitation Department, School of Clinic Medicine & The First Affiliated Hospital of Chengdu Medical College, Chengdu, Sichuan, China; ^6^ Chinese Medicine Rehabilitation Department, Jiahekang Hospital, Luzhou, Sichuan, China; ^7^ Department of Acupuncture, Moxibustion, Tui-Na and Rehabilitation, The Affiliated Traditional Chinese Medicine Hospital of Southwest Medical University, Luzhou, Sichuan, China; ^8^ Center for Traditional Chinese Medicine Prevention and Health Care, Chengdu Integrated TCM & Western Medicine Hospital, Chengdu, Sichuan, China

**Keywords:** inflammatory biomarkers, low back pain, Mendelian randomization, causality, interleukin 6 (IL-6)

In the published article, there was an error in affiliation 1. Instead of “Tui-Na department, Affiliated Hospital of Chengdu University of Traditional Chinese Medicine, Chengdu, Sichuan, China”, it should be “Tui-Na Department, Hospital of Chengdu University of Traditional Chinese Medicine, Chengdu, Sichuan, China”.

The authors apologize for this error and state that this does not change the scientific conclusions of the article in any way. The original article has been updated.

