# Supplementary material for: Assessing the causal relationship between genetically determined inflammatory biomarkers and low back pain risk: a bidirectional two-sample Mendelian randomization study
Source: Front Immunol. 2023 Jul 13;14:1174656. doi: 10.3389/fimmu.2023.1174656 (PMC10372790; doi:10.3389/fimmu.2023.1174656)
Supplement: Supplementary file 1 [file DataSheet_1.docx]

Supplementary Material

# Assessing the Causal Relationship between Genetically Determined Inflammatory Biomarkers and Low Back Pain Risk: A Bidirectional Two-Sample Mendelian Randomization Study

# Li Wenhan,^1^ Lu Qunwen,^2^ Qian Junhui,^2^ Feng Yue,^3^ Luo Jian,^2^ Luo Caigui,^2^ He Wenshan,^4^ Dong Bing,^5^ Liu Huahui,^6*^ Liu Zhongxing,^7*^ Su Chengguo,^3*^

# * Correspondence: Su Chengguo, suchengguo19840804@126.com; Liu Zhongxing, [15931235@qq.com](mailto:15931235@qq.com); Liu Huahui, 442121644@qq.com

# Supplementary Figures


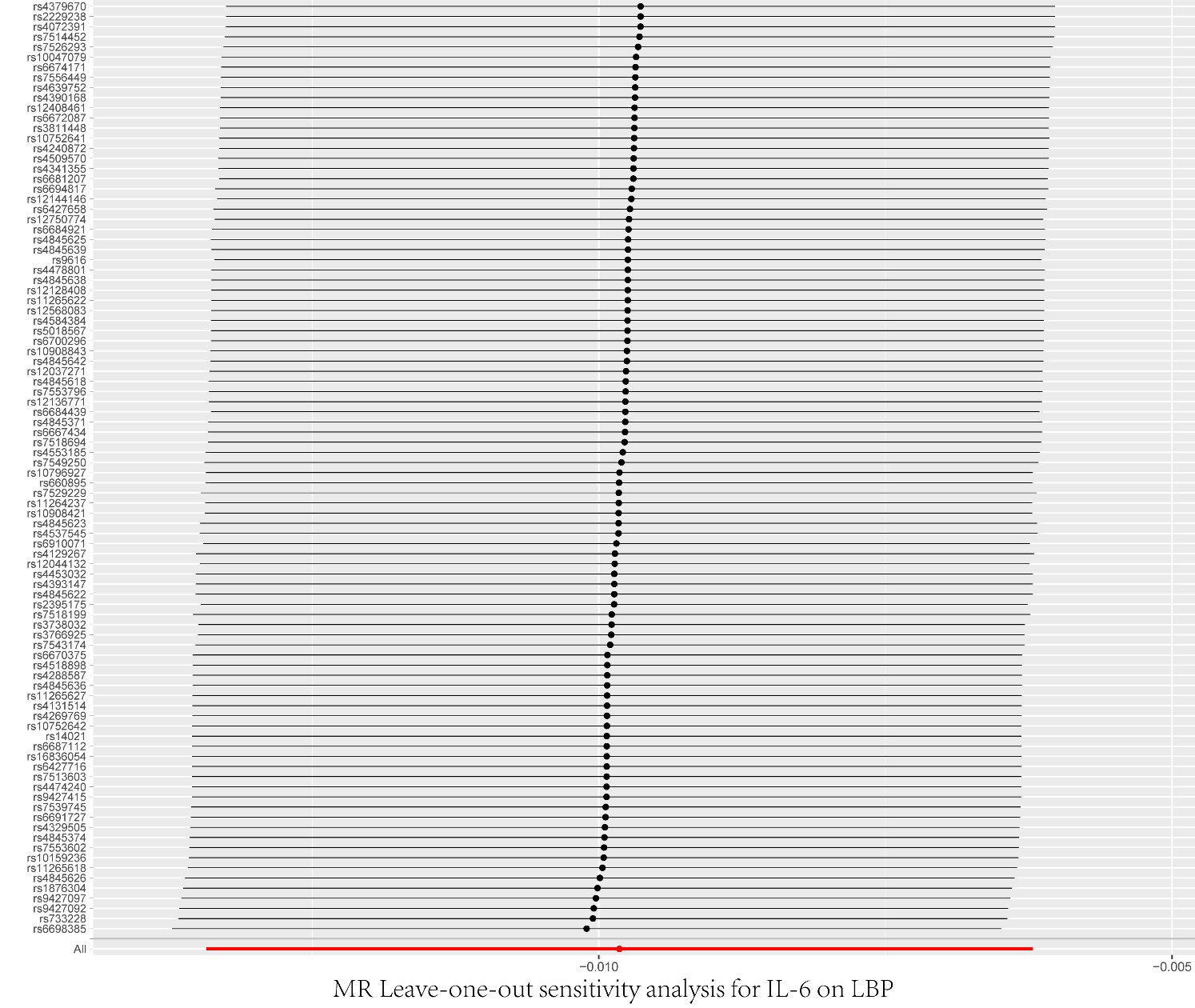


**Supplementary Figure 1.**

Leave-one-out sensitivity analysis for MR analysis. The leave-one-out plots for the causal effects of IL-6 on the risk of LBP. Each row represents the causal effect of the exposure on the outcome estimated in the inverse-variance weighted method after excluding the corresponding genetic variant on the y-axis.

# Supplementary Tables

**Table S1** GWAS data sources used in the present study.

**Table S2** SNP instruments for causal association of CRP with LBP.

**Table S3** SNP instruments for causal association of IL-6rα with LBP.

**Table S4** SNP instruments for causal association of IL-8 with LBP.

**Table S5** SNP instruments for causal association of IL-10 with LBP.

**Table S6** SNP instruments for causal association of LBP with CRP.

**Table S7** SNP instruments for causal association of LBP with IL-6rα.

**Table S8** SNP instruments for causal association of LBP with IL-8.

**Table S9** SNP instruments for causal association of LBP with IL-10.
